# Transfer learning for reaction optimisation: overview of its applications and limits

**DOI:** 10.1039/d6sc02502a

**Published:** 2026-08-19

**Authors:** Aravind Senthil Vel, Maxime Llobet, Thibaud Mabit, François-Xavier Felpin

**Affiliations:** a Nantes Université, CNRS, CEISAM, UMR 6230 F-44000 Nantes France thibaud.mabit@univ-nantes.fr fx.felpin@univ-nantes.fr https://felpin.univ-nantes.fr/

## Abstract

Reaction optimisation remains a critical yet resource-intensive step in chemical synthesis and process development, particularly in complex, multidimensional reaction spaces. Recent advances in automated experimentation and machine-learning-guided optimisation have significantly improved the efficiency of identifying optimal reaction conditions. However, most optimisation campaigns still operate in a cold-start regime, failing to exploit the growing body of historical experimental data. In this Review, we examine the emerging role of transfer learning as a strategy to overcome this limitation by enabling the reuse of knowledge from related reactions or prior optimisation campaigns. We provide a comprehensive overview of transfer learning methodologies applied to reaction optimisation, including multi-task Bayesian optimisation, ensemble approaches, neural-process-based models, hybrid artificial intelligence frameworks, and active transfer learning strategies. Through selected case studies, we highlight how these approaches can accelerate convergence, reduce experimental effort, and improve data efficiency, particularly in automated and flow chemistry platforms. We further discuss key challenges, including the risk of negative transfer, the difficulty of defining reaction similarity, and the need for chemically meaningful representations. Finally, we outline future directions toward knowledge-accumulating autonomous laboratories, where transfer learning enables cumulative chemical learning across reaction systems.

## Abstract 2

Transfer-learning-assisted reaction optimisation accelerates the identification of optimal conditions by incorporating knowledge from prior reactions.

## Graphical abstract

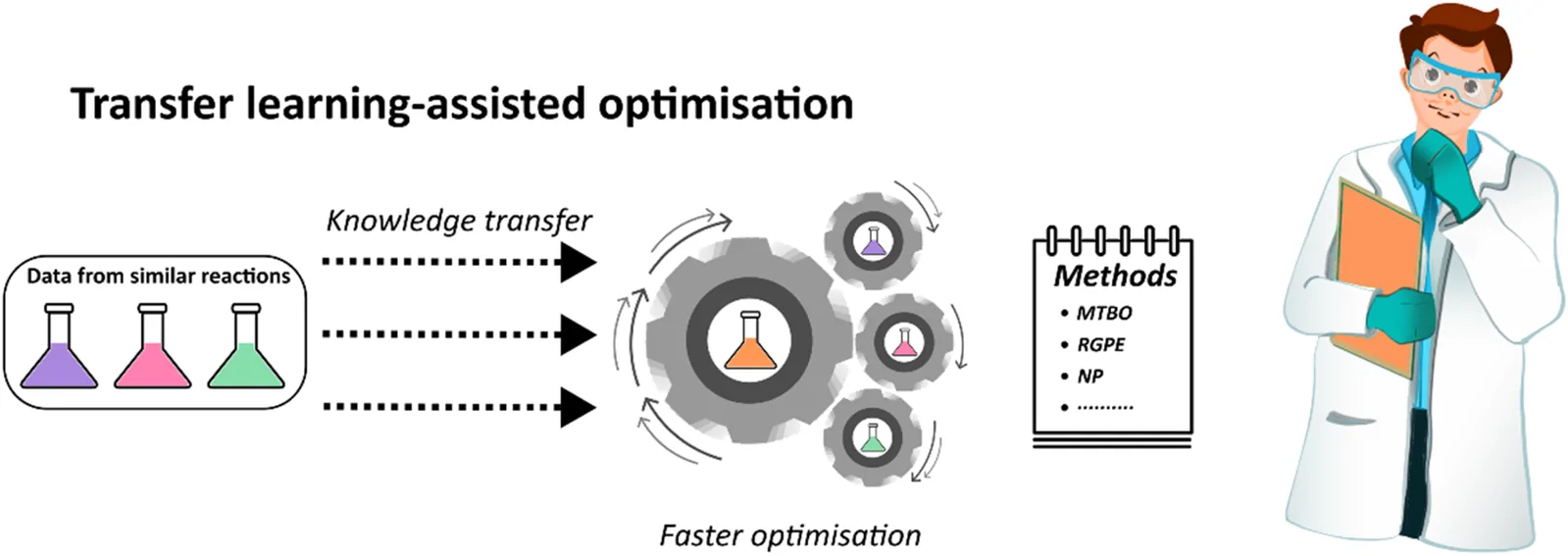

## Introduction

Traditionally, reaction optimisation was performed using simple one-variable-at-a-time (OVAT) or design of experiments (DoE) approaches.^1^ In OVAT strategies, individual parameters such as temperature, concentration, or catalyst loading are varied sequentially while keeping others constant. While intuitive, this method is often inefficient, fails to reveal interactions between variables and highly dependent of the chemist background. DoE methodologies marked an important advance by enabling the simultaneous, statistically guided variation of multiple factors, allowing correlations and optimal regions within the experimental space to be identified. Nevertheless, both approaches can become resource- and time-intensive when applied to complex, multidimensional reaction landscapes. These constraints have driven the emergence of more data-efficient and automated optimisation strategies in recent years.^2^ As automated reaction platforms became more common, particularly with the growing interest in flow chemistry,^3^ algorithms that suggest new experiments based on past experimental outcomes with the explicit goal of optimising reaction performance became more attractive. Such approaches integrate naturally into automated setups and enable reaction optimisation to be carried out in an autonomous manner, commonly referred to as autonomous self-optimising reactor platform.

In the early stages of autonomous reactor development, algorithms such as the Nelder–Mead simplex and steepest-gradient methods were commonly employed.^4–7^ While these methods can be efficient in certain settings, they still exhibit limitations. In particular, they are local search methods and are generally restricted to continuous variables, such as reaction time and temperature.^1,2^

Bayesian optimisation^8^ (BO) later gained attention as an alternative framework for reaction optimisation.^9–11^ BO is a model-based optimisation approach that builds a probabilistic surrogate model from a limited amount of experimental data. An acquisition function, defined on this surrogate model, is then used to select the next interesting experimental condition. After each experiment, the model is updated with the new data, and the procedure is repeated until a satisfactory result is obtained or the experimental budget is reached. BO not only addresses several limitations of earlier optimisation methods but also expands the range of reaction optimisation problems that can be considered. Previously, reaction optimisation was largely confined to identifying a limited set of operating conditions, with the primary objective of maximising reaction yield. In recent years, BO has been used to solve reaction optimisation problems involving multiple objectives.^12–15^ It has also been applied to problems with categorical variables,^10,16,17^ such as catalysts and solvents, and including constraints on reaction parameters.^18–20^

It is important to note that, although innovative methods have been developed in recent years with a focus on reaction optimisation,^19,21^ the underlying approaches were generally not originally developed for chemical applications. These methods already existed and had been used in other fields.^22–25^ BO has long been used for hyperparameter optimisation in machine learning (ML) models before being used in chemistry.^26,27^

One transformative concept that has been used more recently in reaction optimisation is transfer learning (TL), a ML approach in which knowledge gained from related tasks, referred to as source or auxiliary tasks, is exploited to accelerate learning on a new task, referred to as the target task.^28^ This idea is intuitive and closely reflects how humans learn. For example, a person who already knows how to drive a car (source task) can learn to drive a truck (target task) much faster than someone starting from scratch. Chemists also inherently follow this approach by drawing on their experience with related reactions when proposing catalysts, reagents, and reaction conditions for a new but similar reaction.

In ML studies for reaction prediction, models are typically trained on data within a specific domain of reactions. As a result, predictions for reactions outside the training domain are often unreliable.^29^ In such cases, a TL approach improves predictive performance by using knowledge or data from related reaction domains.^30–35^ Similarly, in reaction optimisation, TL can be used to accelerate the search for optimal conditions of a target reaction by leveraging data from similar reactions (Fig. 1). Standard optimisation procedures typically start from zero, commonly referred to as a cold start. This approach is well suited for reactions when no prior data are available, as the model is built solely from the observations of the target reaction. A first pool of experiments is generally selected through latin hypercube samplig (LHS), random sampling or DoE to build a surrogate model. However, many practical scenarios involve reactions with similar substrate classes, reagents, and/or reaction mechanisms. In such cases, instead of starting an optimisation procedure from scratch, data from similar reactions can be used to guide the search towards promising regions, particularly in the early stages, potentially accelerating the optimisation process of the target reaction.

**Fig. 1:**
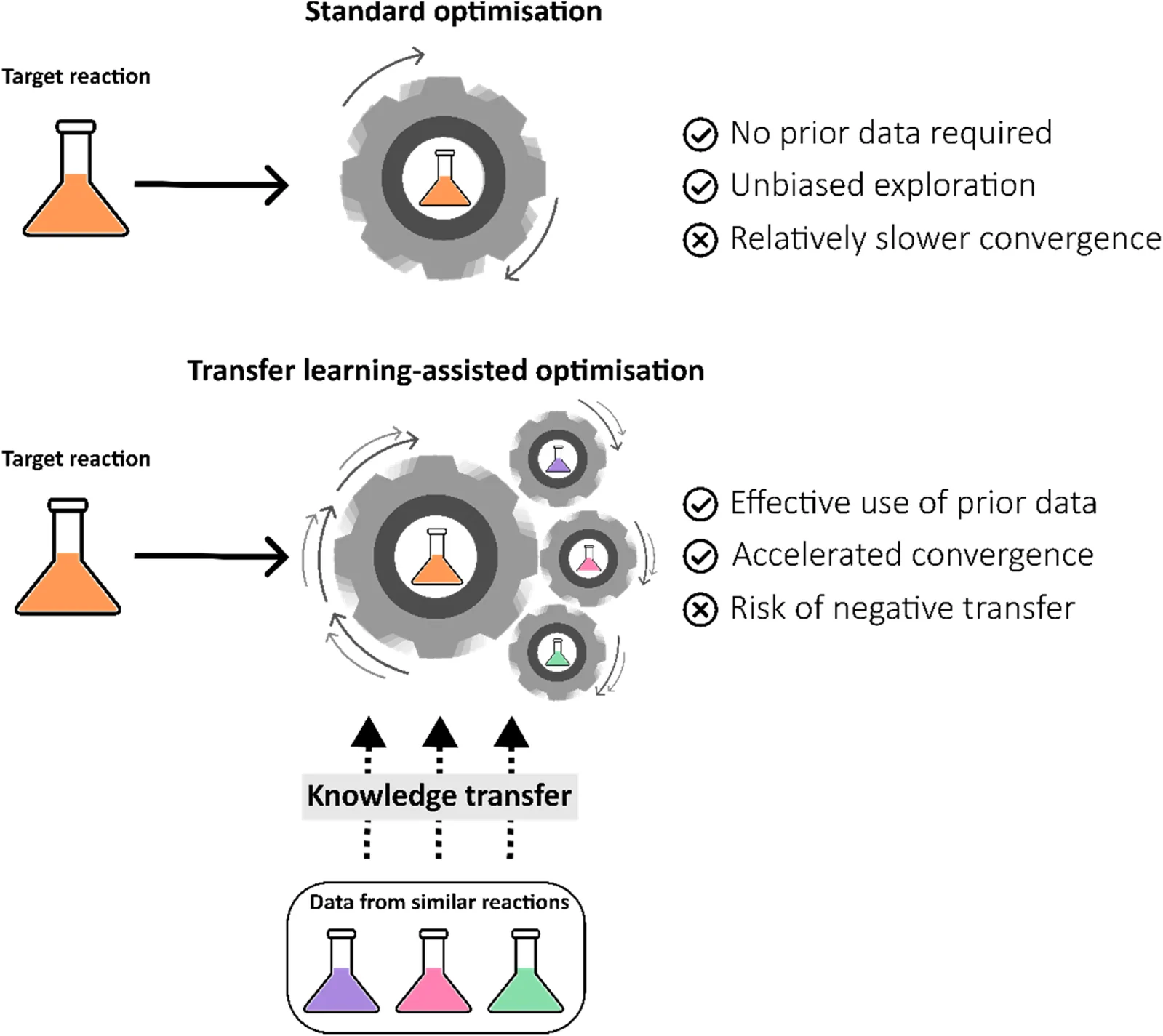
Conceptual illustration of standard optimisation and transfer learning-assisted optimisation.

In this work, we review the methods and practical applications of TL in reaction optimisation. While different TL strategies have been used for reaction prediction, where the focus is on predictive accuracy and often involves large datasets, this work focuses on TL strategies in reaction optimisation, where the objective is to identify the best reaction conditions and the amount of data is relatively limited. Particularly, the focus is on methods applied within BO, though strategies outside BO, such as active transfer learning (ATL), are also discussed.

There are several methods available, particularly within BO, to achieve TL. In the following sections we discuss the methods highliting the practical implementation. For detailed reviews of TL methods in BO not focusing on reaction optimisation the reader can refer to the literature.^36,37^ Given the rapid evolution of this field, we have established an arbitrary cutoff date of March 2026 for the inclusion of publications.

### Explicit modelling

One of the simplest approaches is to explicitly model the existing data, where data from source tasks are directly incorporated into the model by introducing additional parameters to account for task identity. For example, when optimising a target substrate reaction with available data from a related substrate (source) reaction, both source and target tasks can be represented as different levels of a categorical variable. During optimisation, the search is constrained such that new experimental suggestions are proposed only for the target task. In this way, the relationships between variables learned from the source tasks become accessible to the target task.

Faurschou *et al.* reported the use of explicit modelling for TL in carbohydrate chemistry, where data from source reactions are encoded as integers.^38^ In their study, a Gaussian process regressor (GPR) was used within a single-task Bayesian optimisation (STBO) framework. The methodology was applied to optimise the 6-*O*-monobenzoylation and 3,6-*O*-dibenzoylation of several monosaccharides, with experiments conducted on a ChemSpeed Flex robotic synthesis platform that allowed to perform reactions on a 50 mg scale. For each case study, results from preceding optimisation campaigns were incorporated as initial datapoints. Seven variables were optimised, including both continuous and categorical parameters: the base, the benzoylating agent, their respective equivalents, concentration, and the delay between base and acylating agent addition. Temperature and reaction time were fixed, while the starting monosaccharide was treated as a categorical variable to enable transfer learning across substrates.

The first campaign focused on the 6-*O*-monobenzoylation of an β-glucoside and served as the reference dataset for subsequent studies. Comparing the monobenzoylation of glucoside without prior data (Table 1, entry 1) to the corresponding mannoside reaction initialized with glucoside data (Table 1, entry 3) underscores the effectiveness of transfer learning: convergence was achieved after only 23 experiments, compared to 63 in the original campaign. A similar benefit was observed when transferring knowledge to the dibenzoylation of glucoside, for which convergence was reached after 18 iterations (Table 1, entry 2). Further case studies confirmed the effectiveness of explicit modeling, as the optimisation of galactoside derivatives rapidly converged when initialized with prior glucoside and mannoside datapoints.

**Table 1:** Explicit modelling approach for the benzoylation of sugar by Faurschou *et al.*^38^

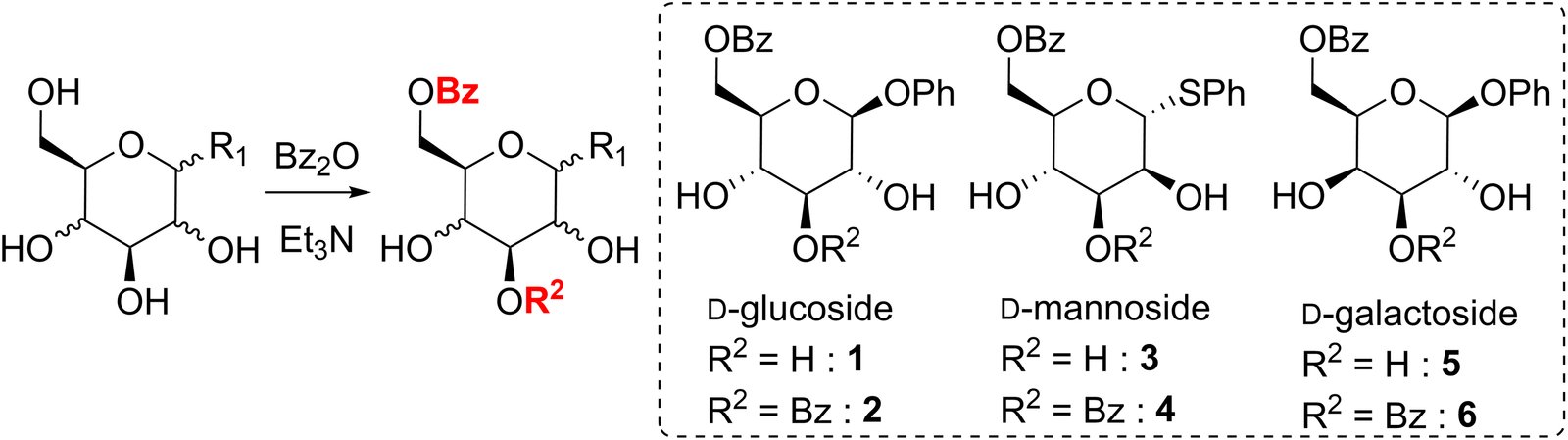					
Entry	Glycoside	Number of source experiments (entry)	Total number of experiments	Number of experiments for highest conv.	Highest conv. (%)
1	1	0	71	63	63
2	2	71 (1)	36	18	67
3^a^	3	71 (1)	47	23	48
4	4	137 (1 + 2 + 3)	24	20	95
5	5	155 (1 + 3 + 6)	25	21	44
6^b^	6	161 (1 + 2 + 3 + 4)	25	17	89

a 2,6-Lutidine and benzoyl chloride were identified as the best base and benzoylating agent.

b *N*,*N*-Diisopropylethylamine was identified as the best base.

In these case studies, regioselectivity was conserved across substrates, with preferential functionalization at O6 (primary alcohol) followed by O3 (equatorial), a selectivity well-known in carbohydrate chemistry. When comparing the structure of each substrate, *e.g.* glucose, mannose, and galactose, they all share the same primary O6 and equatorial O3 substitution. This shared structure likely contributes to a shared reactivity and may explain why the TL performs well in this study. The authors however observed discrepancies between the yields obtained during the BO campaign and those from the manual validation experiments on a 250 mg scale. Despite the fact that it can be consistently rationalized by the changes in scale and the difference in inertness of the reaction medium, it can still affect the conclusions about a TL exclusively based on reaction without taking into account practical experimental considerations such as scale or equipment.

Explicit modelling is effective when the differences between tasks can be encoded in the model, but this is not always straightforward.^39^ An alternative approach is to learn inter-task relationships directly from the data.

### Multi-task Bayesian optimisation

One of the common approach for transfer learning within BO is the use of multi-task Gaussian processes (MTGPs) as the surrogate model.^40^ Gaussian processes (GPs) are a popular choice for the probabilistic surrogate model in BO, especially in chemical reaction optimisation.^41,42^ Within a GP, the kernel (covariance) function, *k*_GP_(*x*, *x*′), defines the similarity between two input points *x* and *x*′. In the single-task setting, this kernel describes how different points are within the same task. In the multi-task GP setting, the intrinsic coregionalization model (ICM) is used to jointly model data from multiple source tasks together with the target task. The covariance function is defined as *k*((*x*,*t*), (*x*′,*t*′)) = *k*_GP_(*x*,*x*′). *k*_T_(*t*,*t*′), where *k*_T_ captures the similarity between tasks *t* and *t*′. The use of MTGPs as the surrogate model within the BO framework gives rise to multi-task Bayesian optimisation (MTBO).^40^ In this setting, the acquisition function is restricted to selecting conditions only for the target task.

There should be no confusion between multi-task Bayesian optimisation, multi-fidelity Bayesian optimisation and multi-objective Bayesian optimisation. In multi-fidelity Bayesian optimisation, a single task is optimised using data of varying accuracy and cost, allowing for a trade-off between evaluation fidelity and resource expenditure.^43^ Correlations between different fidelity levels can be modelled using ICM, similar to those used in MTGPs. Multi-objective Bayesian optimisation aims to optimise multiple objectives (*e.g.*, yield and E-factor) within a single task (*i.e.*, one reaction), whereas the former models one or more objectives across multiple tasks (*i.e.*, different reactions).^15^

Taylor *et al.* reported the first experimental implementation of multi-task Bayesian optimisation MTBO for chemical reaction optimisation, knowledge transfer between related reactions, allowing prior experimental data to accelerate new optimisation campaigns.^44^ The use of MTBO algorithm, integrated into the open-source reaction optimisation package Summit,^45^ was first validated through *in-silico* studies on Suzuki–Miyaura and Buchwald-Hartwing cross couplings.^46^ In a second time, experimental implementation of MTBO was achieved on a series of palladium-catalyzed C–H activation reactions forming pharmaceutically relevant oxindoles (Scheme 1). They employed MTGPs as the surrogate model, that accounts for the data from different but similar reactions, and used q-noisy expected improvement acquisition function. Each iteration updated the surrogate model with online analytical feedback. This closed-loop framework enabled transfer learning and rapid convergence to optimal reaction conditions.

**Scheme 1:**
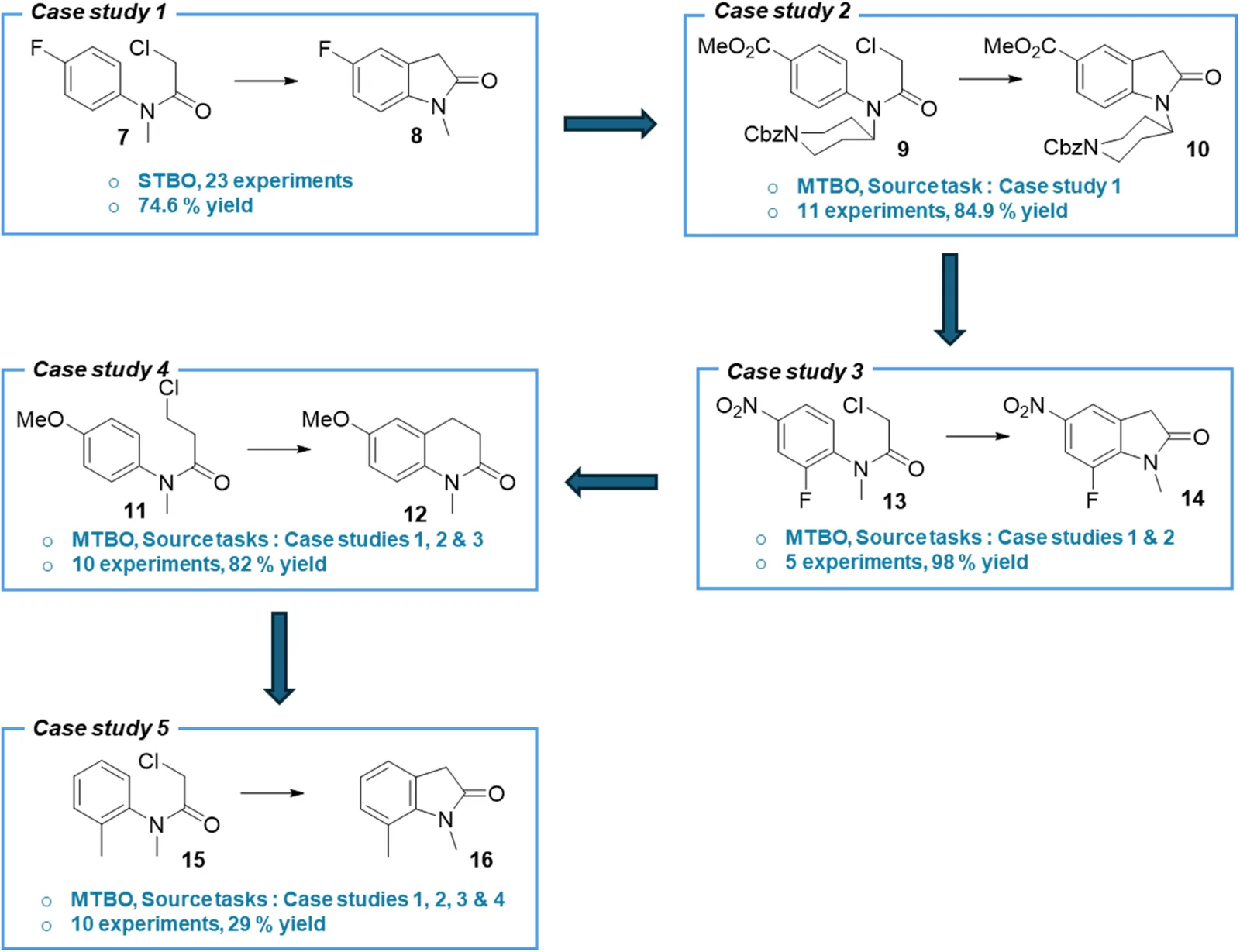
First experimental implementation of MTBO for chemical reaction optimisation by Taylor et *al*.^44^

The results clearly demonstrate the potential of MTBO in accelerating optimisation tasks compared to conventional single-task Bayesian optimisation (STBO) and traditional DoE strategies, with a significant reduction in experimental iterations and material consumption (case studies 2–4, Scheme 1). The methodology performed well when multiple auxiliary/source tasks are available, enabling efficient knowledge transfer across related reactions with the ability to handle mixed categorical and continuous variables. However, both *in silico* and experimental studies showed that MTBO performance strongly depends on the similarity between tasks, and biased behavior can occur when auxiliary datasets are noisy or mechanistically divergent. In such cases, the model may overexploit suboptimal regions of parameter space, slowing convergence. For example, for the *in silico* optimization of 3-chloropyridine and 2-fluoropyridine-3-boronic acid pinacol ester, TL was effective when 2-benzofuranylboronic acid pinacol ester was used as the auxiliary task, whereas detrimental with isoxazole derivative. Performance dropped significantly for substrates with distinct electronic or steric profiles (case study 5, Scheme 1), indicating that broader chemical diversity requires larger and more structured training datasets. When examining case study 3, which proved to be particularly efficient within the TL strategy, the optimal conditions were highly similar to those of case study 1, explaining the rapid convergence toward high-yielding reactions. For case study 5, the algorithm identified a maximum in the search space under conditions similar to those of case study 2 (Table 2). However, the aryl moiety differs electronically, which may explain why the yield in case study 5 remains moderate and why the algorithm may be trapped in a local maximum. This further supports the idea of enriching the system with appropriate chemical descriptors. Further development may be needed to improve task classification, model robustness, and generalization across diverse chemistries.

**Table 2:** Optimal conditions for chemical reaction optimisation by Taylor et *al*.^44^

Case study	Solvent	Ligand	Time (min)	Temp. (°C)	mol%
1	NMP	Xphos	60	85	8
2	ACN	JohnPhos	28	127	5
3	NMP	XPhos	60	95	9
4	DMSO	DPEPhos	60	150	10
5	ACN	JohnPhos	37	150	6

Using an optimised amide coupling reaction as an auxiliary dataset, Wagner *et al.* also applied a MTBO framework to three transfer scenarios within a self-optimising flow reactor (Scheme 2).^47^ These included changing the coupling reagent, replacing the substrate with a significantly less nucleophilic amine, and modifying both the substrate and the reagent.

**Scheme 2:**
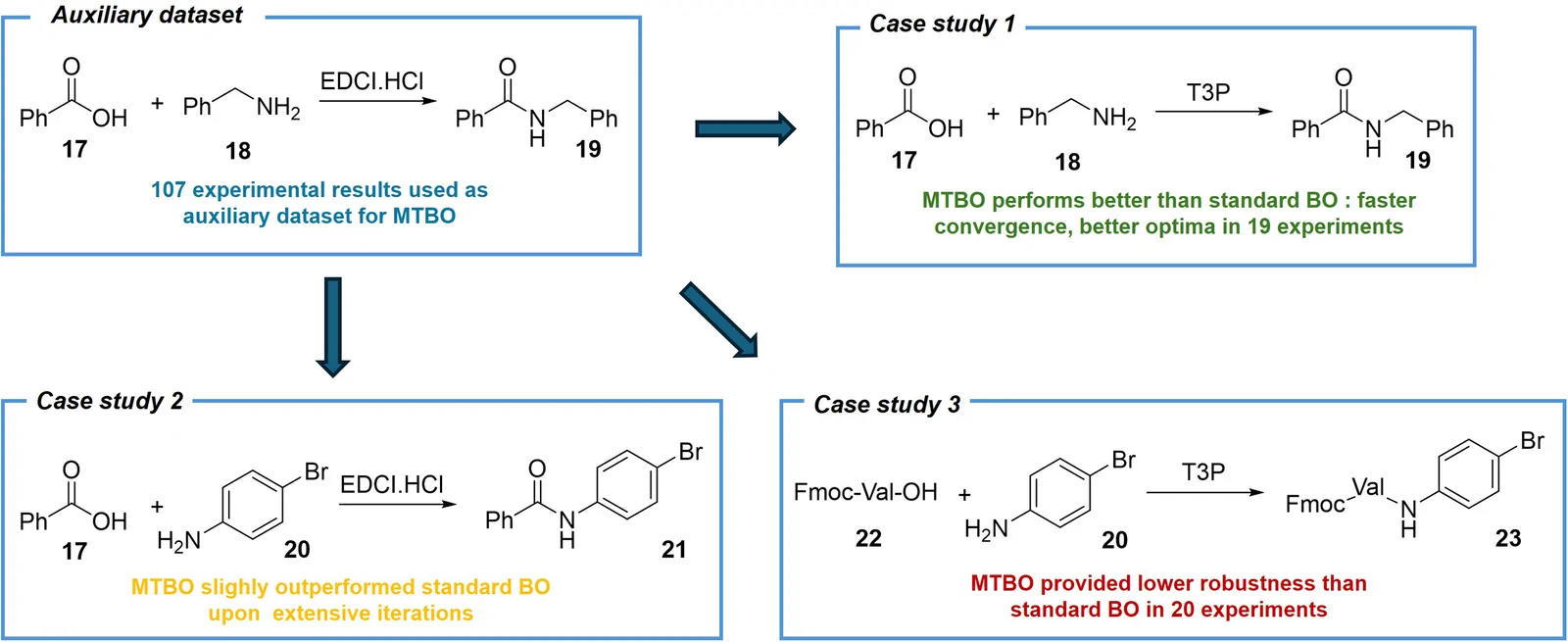
MTBO approach for amide coupling reactions by Wagner et *al.*^47^

When applied to chemically similar systems (*e.g.*, changing the coupling agent EDCI·HCl for T3P), MTBO markedly accelerated convergence, rapidly identifying a satisfactory optimum within a limited experimental budget (case study 1, Scheme 2). This demonstrates the efficient reuse of prior experimental data to reduce material consumption and optimisation time when reaction mechanisms and reactivity profiles are closely related. For reactions involving substantially lower reactivity (electron-poor anilines 20), the auxiliary data set biased early experiments toward unproductive regions of the design space, leading to lower early-stage performance compared to standard BO (case study 2, Scheme 2). Nevertheless, MTBO ultimately reached comparable or superior optima after sufficient iterations. In the third scenario investigated by the authors where both coupling substrates and coupling agent were modified, standard Bayesian optimisation demonstrated stable performance, yielding progressively improved results, while the MTBO approach sampled a substantial number of low-yielding conditions (case study 3, Scheme 2).

These results illustrate that in the absence of chemically informed criteria for selecting or weighting auxiliary data sets, the MTBO approach is sensitive to task mismatch and naïve knowledge transfer may be detrimental in cases of poor reactivity overlap. The authors consequently emphasized the need for reactivity descriptors or similarity metrics to guide task selection.

MTBO suffers from scalability issues arising from the use of a single GP model to jointly represent all source and target data. Since GP inference scales cubically with the number of data points, this approach become computationally prohibitive as the number of source tasks and the amount of data per task grow. This limitation is addressed by the ranking-weighted gaussian process ensemble (RGPE) approach, in which separate GP models are fitted independently for each source and target task.

### Ranking-weighted Gaussian process ensemble

In RGPE, separate GP models are fitted independently for each source and target task and combined as a weighted ensemble.^37^ Unlike the methods described above, where the entire joint model must be refitted each time new target task data are collected, in RGPE only the target task model is refitted while source task models remain fixed. The weight assigned to each model is determined by how well it ranks the observed target task data. At the beginning of optimisation, source task models receive higher weights due to the limited data available for the target task. As more target task data are collected, the target model naturally becomes dominant and the contribution of source task models is gradually reduced, allowing the method to benefit from prior knowledge while limiting the influence of unrelated tasks. To our knowledge, the application of RGPE in chemical reaction optimisation has been demonstrated only *in silico,* using datasets from Pd-catalyzed Buchwald–Hartwig amination reactions,^48^ while its translation to real experimental campaigns remains to be reported.

### Neural processes

Neural processes (NPs)^49^ offer a computationally efficient alternative to GPs as they scale linearly with the number of data points. Unlike GPs, which rely on predefined kernel functions to capture relationships between data points, NPs use a neural network-based formulation, eliminating the need to select a suitable kernel. A NP consists of three main components: an encoder, an aggregator, and a decoder. The encoder processes each observed input–output pair through a neural network to produce a learned representation. The aggregator combines these representations into a single summary that captures the shared structure across the observed data. The decoder then takes this summary along with new target inputs to predict the corresponding outputs with uncertainty estimates. During training, the model learns across multiple tasks, enabling it to extract transferable patterns from observed data and apply them to new tasks.

Guo *et al.* use NP in place of GP within a multi-objective BO framework to scale up a flow synthesis process from laboratory to industrial scale (Fig. 2).^50^ The process studied is the synthesis of *O*-methylisourea by nucleophilic addition of methanol on the cyanamide in acidic conditions. The strategy chosen was to optimise two objectives: productivity and E-Factor, over 3 continuous variables: reaction time, temperature and equivalence of H_2_SO_4_.

**Fig. 2:**
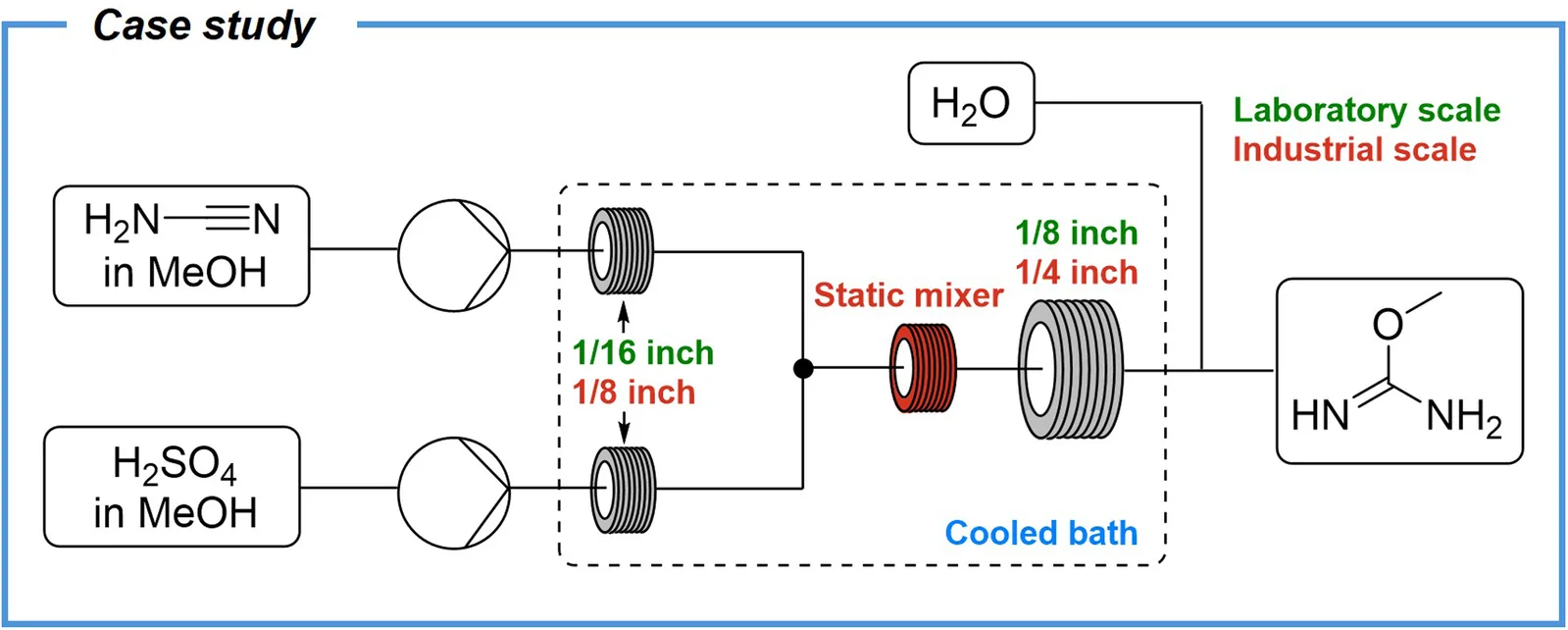
Flow setups at the two different scales used for knowledge transfer by Guo et *al.*^50^

During laboratory-scale development, the reaction conditions were optimised using an in-house multi-objective Bayesian optimisation (MOBO) framework called FlowBO. Similar to many BO algorithms, FlowBO uses GPs to generate its surrogate model and noisy parallel expected hypervolume improvement (qNEHVI) as acquisition function. In contrast to conventional single-stage optimisation strategies, the optimisation was conducted in two successive rounds. In the first round, the ranges of the reaction parameters were restricted based on preliminary experimental investigations. Thereafter, a second optimisation round was performed in which selected parameter bounds were expanded according to the trends and insights obtained from the first round.

To facilitate rapid and robust scale-up, Guo *et al.* implemented a transfer learning approach using NPs instead of GP within the BO framework. In order to adapt the model created on the previous tasks to the new industrial-scale process, only three new experimental measurements were conducted.

In this strategy, the BO algorithm exploited the operating conditions that had yielded promising results during preliminary development at the laboratory scale. However, due to the larger reactor size, the measured productivity values were significantly higher than those obtained in previous campaigns. It emphasizes that the application of TL to process chemistry should consider not only the reaction but also the experimental context. A more process-oriented featurization, incorporating hardware-related descriptors,^51^ would enrich reaction representations, could improve TL performance in scale-up projects and prevent negative TL. Furthermore, the limited number of new experimental observations, all from relatively low-yield regions, has potentially limited the model's ability to accurately capture the broader response landscape. As a result, the optimisation process focused on a localized region of the design space, reducing the scope of exploration and limiting the conclusions about the overall effectiveness and robustness of the optimisation approach.

A limitation of standard NPs is that the aggregator assigns equal weight to all observed data points regardless of their relevance to the target input, which can lead to poor predictive performance. Attentive neural processes (ANPs) address this by introducing an attention mechanism, allowing each target input to selectively focus on the most relevant observed data, improving prediction accuracy.^52^

Hickman *et al.* used ANPs, a variant of the NPs, to incorporate an additional constraint term into the acquisition function, alongside the acquisition function based solely on the target task.^53^ They developed SeMOpt, a transfer learning algorithm based on BoTorch.^54^ The transfer was achieved through a compound acquisition function. The acquisition function derived from the ANP model, trained on both source and target tasks, was combined with that of the target model, which was trained only on the target task and based on GP. This biased the search towards regions identified as promising in the source tasks. As the optimisation progressed, the influence of the ANP acquisition term was gradually reduced, allowing the method to naturally ignore source information when it becomes less relevant.

The performance of SeMOpt was first evaluated on a benchmark study using the Reizman dataset,^55^ which consists of cross-coupling reactions simulated through kinetic modeling. Five simulation cases were considered, each defined by four variables, both continuous and categorical: catalyst, temperature, reaction time, and catalyst concentration. Each reaction optimisation task was performed using the remaining four case studies as source domains. In all scenarios, SeMOpt outperformed baseline single-task Bayesian optimisation methods as well as meta-learning algorithms.

The algorithm was subsequently applied to the *in silico* optimisation of a Buchwald–Hartwig coupling reaction. The dataset introduced by Ahneman *et al.*^56^ comprises couplings between 4-methylaniline and 15 different aryl halides. The reaction space included 23 additives, 4 catalysts, and 3 bases, resulting in 276 possible reaction conditions for each coupling partner. For the optimisation of a given electrophile, the objective was to identify the optimal combination of catalyst, additive, and base, leading to the best yield and using the results obtained for the other 14 halides as a pre-training dataset. SeMOpt-based approaches were able to identify the top two reaction conditions within 11 or fewer evaluations, whereas standard BO required between 17 and 28 iterations, clearly demonstrating the efficiency of the transfer learning strategy (Table 3).

**Table 3:** Buchwald–Hartwig *in silico* optimisation case study^53^

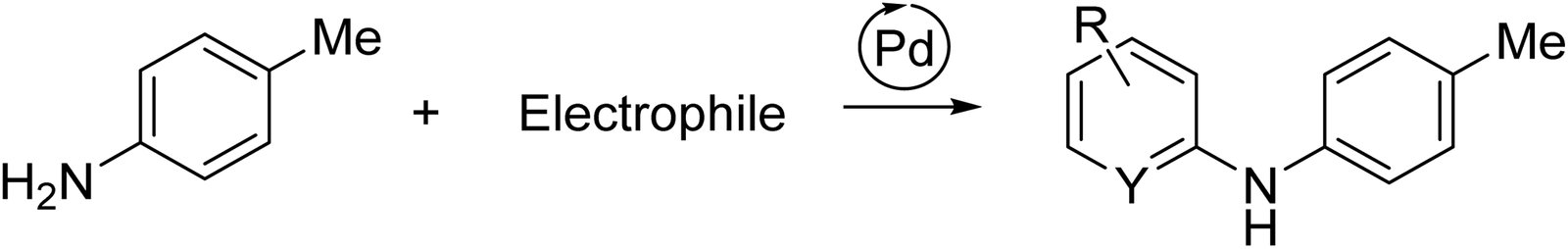				
Entry	Electrophile	*n*_top-2SeMOpt_ a	*n*_top-2BO_ b	*n*_top-2Rand_ c
1	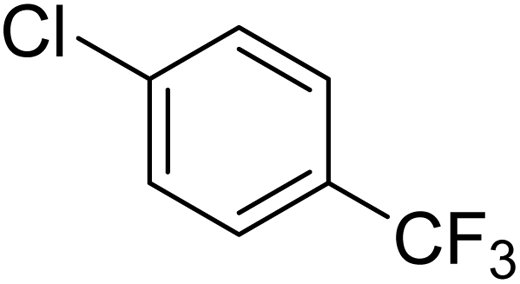	11.00	17.07	79.47
2	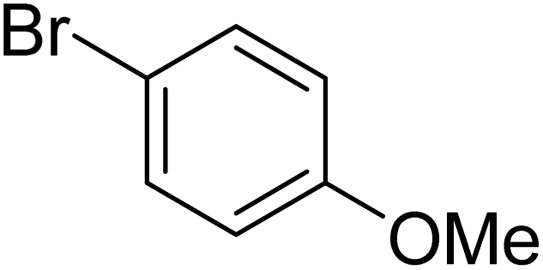	6.60	25.73	91.53
3	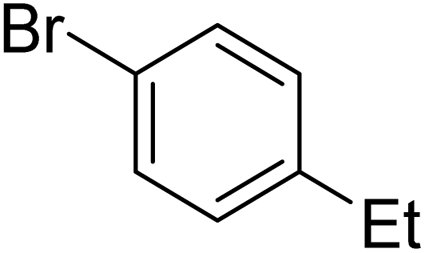	9.88	28.15	99.92
4	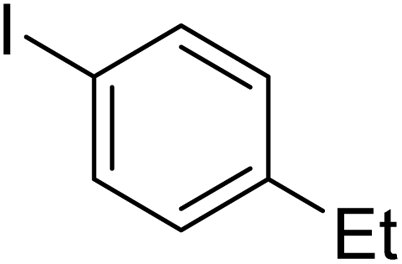	9.18	26.57	91.25
5	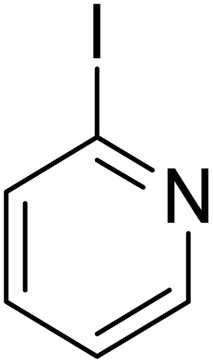	9.72	25.08	104.95

a Average number of experiments to identify top-2 yield over 40 runs with SeMOpt.

b Average number of experiments to identify top-2 yield over 40 runs with standard BO implemented with GPyOpt.

c Average number of experiments to identify top-2 yield over 40 runs with random search. Y = CH, N.

For relatively simple optimisation tasks, characterized by a high proportion of high-yielding conditions (Fig. 3, aryl 3 and 4), SeMOpt showed a slighter improvement over other methods. In contrast, for more challenging optimisation problems, where only a limited number of conditions lead to high yields, SeMOpt showed a significant advantage, converging more efficiently toward optimal solutions (Fig. 3, aryl 1 and 2). In these cases, TL–based approach appears particularly powerful, needing less than 3 equivalents to reach the top–5 yields.

**Fig. 3:**
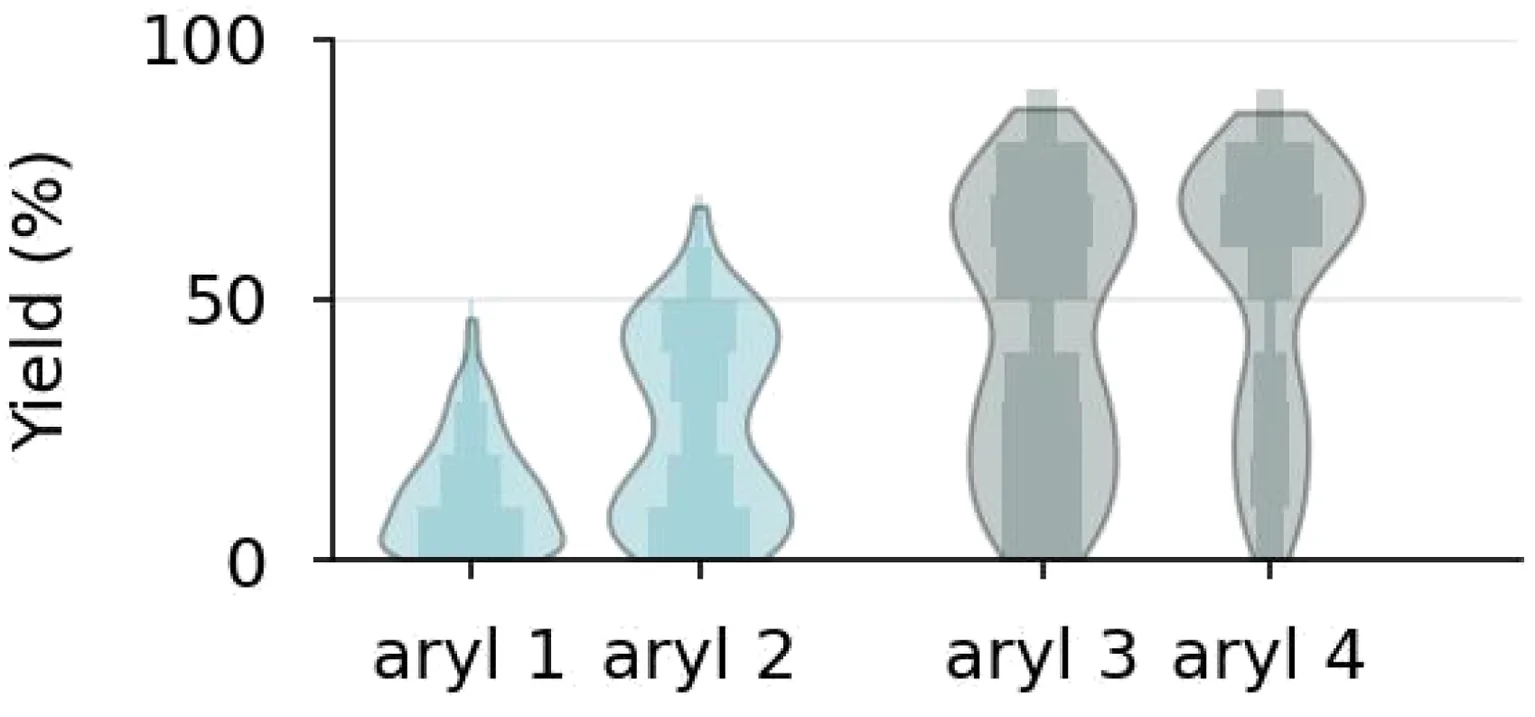
Yield distribution of coupling reactions with different aryl halides by Ahneman et *al.*

These results are consistent with the notion that transfer learning through acquisition function tuning is particularly beneficial in complex optimisation landscapes, where prior knowledge can meaningfully guide exploration. A real-world reaction optimisation, is not described by the authors in the paper and would substantially strengthen the conclusions (authors very recently published preprint about the application of SeMOpt in a substrate-adaptive decarboxylative olefination reaction optimisation)^57^ While the algorithm is designed to handle uninformative source data, the authors note that selection of source datasets guided by intuition could further improve performance during early optimisation stages. In the case study, the auxiliary tasks cover a broad yet focused range of cross-coupling reactivities, including different halides as well as various substitutions on the aromatic ring. Thus, the model can efficiently extrapolate and apply this knowledge to the target task. In addition, all source and target tasks originate from a single database, ensuring homogeneity in experimental conditions, favoring effective knowledge transfer and limiting the risk of negative TL. To further strengthen the approach, an expert-driven featurization may enhance the effective use of transferred information.

In the approaches discussed above, experimental data from previously studied similar reactions are used to accelerate optimisation of a new reaction within the BO framework. In contrast, some hybrid approaches combine BO with models trained on large reaction datasets.

### Hybrid methods

Kwon *et al.* introduced a hybrid-type dynamic optimisation (HDO) framework that combines BO with graph neural networks (GNNs), specifically implemented in the form of message passing neural networks (MPNNs), to accelerate the identification of high-yielding reaction conditions while mitigating the cold-start problem inherent to BO.^58^ In this approach, reactions are represented as molecular graphs, and the MPNN learns structure–reactivity relationships by iteratively exchanging information between bonded atoms to prioritize promising reaction conditions. The message-passing neural network was trained on over one million reactions extracted from the Reaxys^59^ database to predict suitable catalysts, ligands, bases, and solvents directly from reaction graphs enabling early-stage exploitation, while BO (GP surrogate model with UCB acquisition function) corrects model bias and promotes exploration as experimental data accumulate. The HDO approach was benchmarked against random search, standard BO, random forest models, message-passing neural networks alone, and human experts across multiple reaction classes (Suzuki–Miyaura, Buchwald–Hartwig, arylation, Ullmann, and Chan–Lam couplings).

Across both data-driven benchmarks and laboratory experiments, HDO consistently identified high-yielding conditions and better improvement rates than random search, random forest models, message-passing neural networks. HDO also consistently demonstrates faster early-stage convergence and higher initial yields than standard BO under identical experimental budgets, however, as experimental data accumulate, the performance gap between HDO and conventional BO narrows. Standard BO progressively corrects its surrogate model based solely on experimental observations, while HDO increasingly relies on BO to compensate for biases inherited from the pretrained neural network. Consequently, both approaches tend to reach comparable optima at later stages of optimisation, with the primary advantage of HDO residing in accelerated early discovery rather than superior final performance. This comparison highlights HDO as advantageous in low-budget or time-constrained optimisation scenarios, whereas conventional BO remains robust and data-efficient in the absence of reliable prior knowledge.

A key limitation is the strong dependence on large and representative training datasets, with reduced performance for underrepresented reaction classes. Furthermore, the need for extensive data to train reaction-specific GNNs may restrict transferability to less-studied chemistries. Recently, a hybrid approach based on large language models has been proposed, termed BORA (Bayesian Optimisation Research Assistant).^60,61^ Large language models (LLMs) are trained on large collections of text and use information provided in plain text form.^62^ BORA leverages the domain knowledge and reasoning capability of these models, guided by user provided contextual descriptions of the optimisation problem. The method follows an adaptive heuristic strategy in which the search alternates between BO, LLM based suggestions, and a combination of both. In this framework, the LLM can provide warm start suggestions, helping to mitigate the lack of inherent domain knowledge in BO. The advantages of the method have been demonstrated through benchmark studies on individual optimisation problems. It would be interesting to explore this approach in the context of TL, where data and knowledge from related reactions could be provided in plain text form.

While the methods described in the previous sections focus on TL strategies within BO for reaction optimisation, TL has also been applied in active learning frameworks which aim to improve the predictive performance of the model, particularly in identifying suitable reaction conditions.

### Active transfer learning

Shim *et al.* combined transfer learning and active learning, referred to as active transfer learning (ATL),^63^ to optimise the nickel-catalyzed formal coupling of amines and carboxylic acids to form secondary alkyl bonds.^64^ Unlike BO, which focuses on fine-tuning reaction conditions, ATL operates, here, as a classifier to predict whether a reaction outcome would improve over the previous best result, making it particularly suited to identifying better sets of reagents amongst large categorical variable spaces. A random forest algorithm was trained to predict reaction outcomes, with the objective function defined as a binary classification determining whether a given reaction condition could outperform previously observed datapoints. Initially, the model consisted of an ensemble of 100 models built using source reaction data. At each iteration, the current model was used to rank untested target reactions, and the highest ranked candidates were selected for the next experimental batch. After obtaining the new experimental results, additional decision trees were trained on the newly collected target data and were added to the existing model. In this way, the model gradually adapted to the target reaction space while retaining knowledge from the source domain. The reaction space consisted of 5 nickel precatalysts, 29 ligands, 14 additives, and 9 solvents, corresponding to 18 270 possible reaction conditions. Chemical components were either one-hot encoded or represented using physicochemical descriptors. The source dataset used for transfer learning comprised 163 coupling reactions involving triphenylpyridinium (TPP^+^) salts as activated amines and alanine- or proline-derived *N*-hydroxyphthalimides (NHPI). Across all case studies, the algorithm identified reaction conditions that furnished higher yields than the control experiments provided in the source dataset (Table 4).

**Table 4:** RF-based Active Transfer Learning for the formal coupling between carboxylic acids and amines by Shim *et al.*^64^

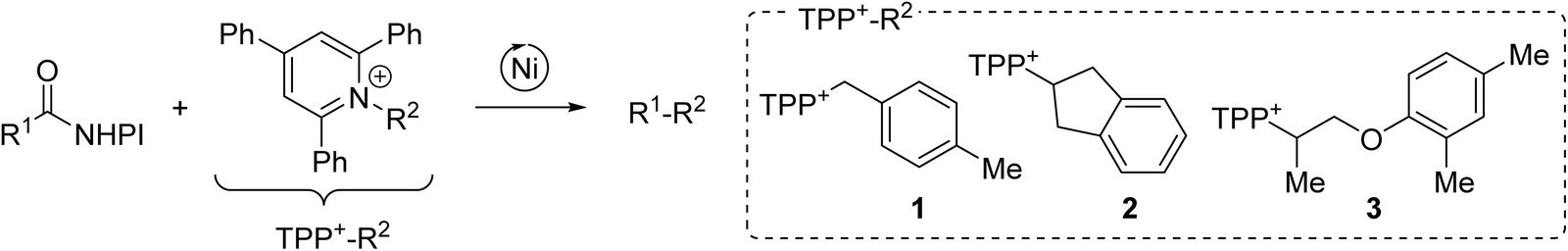							
Case study	R^1^	TPP^+^-R^2^	Run	Number of source experiments	Highest conv from previous experiments	Number of experiments per run	Highest conv. (%)
1	*N*-Boc-Ala	1	1	163	55	3	50
			2	166	55	4	65
2	*N*-Boc-Pro	2	1	163	37	6	33
			2	169	37	6	33
			3	175	37	6	48
3	*N*-Boc-Ala	2	1	163	13	6	20
			2	169	20	6	25
			3	175	25	6	37
4	*N*-Boc-Pro	3	1	163	7	24	9
			2	187	9	24	9
			3	211	9	24	30

However, in case study 1, the optimal conditions were not directly proposed by the model but were instead inferred through human analysis of previously recommended experiments. For case studies 2, 3, and 4, the algorithm successfully identified improved conditions, increasing yields by +15%, +17%, and +21% over 12, 18, and 72 experiments, respectively. In case study 3, a comparative analysis was conducted to assess the efficiency of different machine learning strategies. Active transfer learning proved to be the most effective, reaching the optimum by the fourth experimental batch, whereas transfer learning and Bayesian optimisation evaluated the same condition in the sixth iteration, and random exploration required nine iterations.

Across all studies, the source model narrowed the ligand search space from 29 candidates to a single ligand, thereby substantially reducing experimental effort to 630 potential reaction conditions. While the objective of yield improvement was achieved in all cases, the predictive accuracy of the model appears moderate, as several iterations comprising multiple experiments were required before improvement of the yield. Further enhancements could be achieved through more sophisticated featurization strategies that better incorporate chemical knowledge into molecular representations. Future perspectives include extending the active transfer learning approach to regression tasks instead of binary classification, as a current limitation of the approach lies in its inability to quantify the degree of yield improvements.

## Discussions

Even with the availability of numerous ways to achieve TL, and with some successful applications in reaction optimisation, it remains unclear how best to exploit prior knowledge and, crucially, when such knowledge should not be used. In transfer learning, the effectiveness is not always guaranteed and negative transfer^65^ can occur, where information from source tasks biases the optimisation process and leads to sub-optimal solutions that are worse than those obtained with standard BO without knowledge transfer. This behavior has arisen, for example, in certain *in silico* studies of Suzuki–Miyaura and Buchwald–Hartwig cross-coupling reactions described by Taylor *et al.*^44^ In real experimental studies, such negative transfer may go unnoticed unless the transfer learning approach is explicitly compared with classical BO.^50^

Negative transfer can arise when knowledge is transferred from unrelated tasks. However, it is not always clear how to define whether two reactions are sufficiently similar to justify knowledge transfer. Similarity may arise from comparable reaction mechanisms, similar reagents or substrate classes, or a combination of these factors, and the degree of similarity can vary. Moreover, it is unclear whether such similarity can be reliably identified and measured *a priori*.^35,63^ In the studies discussed above, reactions similarities were not explicitly analysed or considered. A systematic assessment of reaction similarity could help clarify when transfer learning methods are appropriate and when they are likely to fail, thereby guiding the choice of optimisation strategy depending on the relatedness of the tasks.

A way to specify or represent chemical similarity is to use chemical descriptors that capture the intrinsic reactivity of the different reaction components, providing a more informative representation for optimisation. Several studies have explored the use of such chemical descriptors in reaction optimisation campaigns.^66–69^ The use of physicochemical descriptors makes it possible to transform categorical variables into continuous representations and to predict the influence of a ligand or solvent based on their molecular properties, even when the available experimental data originate from different reactions. In explicit modelling approach, representing categorical variables through descriptors may capture similarity more effectively than encoding them using one-hot vectors or integers. Although beyond the scope of this review, we highlight that in a recent study on the reaction encoding based on the mechanism, Gallarati *et al.*^70^ identified specific descriptors to predict the outcome of nickel cross-coupling reactions between styrene oxides and iodoarenes. Through knowledge transfer, these mechanism-informed features were then incorporated into the BO of the reaction enantioselectivity of a coupling between *α*-oxy radicals derived from benzyl acetals and iodoarenes, representing an efficient way to capture the reagent similarity applied in two different reactions.

However, representing chemical similarity through descriptors does not necessarily guarantee similarity in reaction performance or in the underlying reaction behaviour. The RGPE method explicitly addresses this limitation, where the similarity between tasks is learned directly from the data, without the need to specify it *a priori*. It includes a mechanism that down weights information from unrelated source tasks, allowing the optimisation to revert to standard BO when the source and target tasks are not sufficiently related.^37^ This avoids an additional similarity analysis step and reduces the risk of negative transfer. It is worth noting that Hickman *et al.*, using ANP, demonstrated the effectiveness of their method in the presence of unrelated tasks.^53^

At this stage, comparisons must be established across three critical dimensions: (1) convergence rate *versus* final objective yield, (2) source *versus* auxiliary task performance, and (3) classical Bayesian optimization *versus* TL approaches. The current major advantage of transfer learning is not to consistently deliver superior final objective scores as the observed objective values often remain comparable to those achieved without TL. However, the main benefit of TL approaches lies in convergence speed, that is the efficiency with which optimal or near-optimal experiments are identified. This distinction demands a two-part discussion. The critical question becomes: does optimization benefit from chemically aware initial experiments, *i.e.* does the target task converges faster to auxiliary task ? Faurschou *et al.*^38^ demonstrates the power of TL by showing auxiliary tasks converging 63–73% faster than the source task. Taylor *et al.*^44^ reports similar findings, with auxiliary convergence 52–72% faster than the source. Thus, when transfer stems from readily accessible source tasks (such as reactions using available reagents), TL appears particularly appealing. The second key question compares classical Bayesian optimization with transfer learning approaches on the same task. Wagner *et al.*^47^ reveal that convergence rates depend heavily on reaction type, underscoring the critical and still unsolved need to rationalize when transfer learning delivers genuine gains, and when it does not. Shim *et al.*,^64^ conversely, provide more direct evidence for transfer learning's effectiveness. In their comparative study of random search, classical BO, and TL demonstrates that transfer learning improves both the objective score and convergence rate: optimal conditions were identified in just 4 batches of experiments using transfer learning, compared to 6 batches for classical BO and 9 for random search. The same behavior was observed by Hickman *et al.*^53^ with significant improvement of TL compared to others to reach the top-2 conditions. This acceleration illustrates that while reaction-dependent variability remains a concern, transfer learning can deliver substantial practical advantages when applied thoughtfully.

Given the substantial benefits that transfer learning can offer for chemical reaction optimization, we provide practical guidance for researchers seeking to implement these approaches. To this end, Table 5 compares the principal transfer learning strategies discussed in this Review, highlighting their respective requirements, strengths, and limitations. For each approach, we summarize the type of source data required, the underlying model, the mechanism of knowledge transfer, the treatment of task similarity, and the level of experimental validation achieved.

**Table 5:** Comparison of transfer learning methods for reaction optimisation

Method	Source data	Model	Knowledge tranfer	Task similarity	Application
Explicit modelling	Previous optimisation campaigns or reaction data of related reactions	GP^a^	Source and target data are combined, with each identified by a task variable	Defined (encoded) by the user	Applied experimentally to the optimisation of carbohydrate benzoylation reactions^38^
MTBO	Previous optimisation campaigns or reaction data of related reactions	Multi-task GP	Source and target tasks are modelled jointly using a shared kernel	Learned from the data as correlations between tasks	Applied experimentally to C–H activation^46^ and amide-coupling reactions^47^
RGPE	Previous optimisation campaigns or reaction data of related reactions	Ensemble of GPs	One GP is built for each task and predictions are weighted	Learned by ranking how well each source model predicts the target results	*In silico* study^48^
NP	Previous optimisation campaigns or reaction data of related reactions	NP	Shared patterns are learned and adapted using target experiments	Learned from common patterns across the training tasks	Applied to transfer between laboratory- and industrial-scale flow processes^50^
Hybrid	Large reaction database	GNN + GP	The GNN, pretrained on a large reaction database, proposes early conditions; BO then refines the search as experimental data accumulate	Inferred from molecular structures and reaction patterns	Demonstrated experimentally across several coupling-reaction classes^58^
Hybrid	Large collection of text (for LLM training) and user provided information	LLM + GP	The LLM suggests conditions from its domain knowledge and the user-provided problem description, alternating with BO suggestions	Not explicitly calculated; depends on the provided context and the model's learned knowledge	*In silico* study^61^
ATL	Reaction datasets of related reactions	Random forest ensemble	A source-trained classifier ranks target conditions; target-trained trees are added after each round	Assumed when selecting related source reactions	Applied experimentally to nickel-catalysed coupling reactions involving large categorical reagent spaces^64^

a The approach is model-agnostic and compatible with any surrogate that can take the task identity as an input variable; a GP is indicated here as it was used in the reported application^38^ and is the most commonly used surrogate in BO.

Recently, with the increasing adoption of BO, several open-source BO packages, tailored for reaction optimisation, have been developed and maintained by both academic and industrial communities (Table 6). Examples include EDBO+,^71^ BOFIRE,^72^ ProcessOptimiser,^73^ BayBE,^39^ and Atlas.^48^ Many of these packages include transfer learning features, and here we outline the methods available within them. It should be noted that these packages are rapidly evolving, and readers are encouraged to consult the respective documentation for the most up-to-date features.

**Table 6:** Open-source BO packages integrating transfer learning methods

Package	TL methods
BOFIRE^72^	MTBO
BayBE^39^	Explicit modelling, MTBO
ProcessOptimiser^73^	Explicit modelling
Atlas^48^	RGPE, deep kernel transfer

## Conclusion

Transfer learning is emerging as a powerful paradigm for overcoming one of the central limitations of algorithm-driven reaction optimisation: the need to repeatedly “start from scratch” despite the vast amount of chemical knowledge already available. By enabling the reuse of historical data, mechanistic intuition, or related optimisation campaigns, transfer learning offers a route toward significantly reducing experimental burden, accelerating convergence, and improving sustainability in chemical development.

The studies discussed in this review demonstrate that knowledge transfer can be implemented through multiple complementary strategies, including explicit modeling, multi-task Bayesian optimisationranking-weighted Gaussian process ensemble, neural process–based learning, hybrid frameworks, and active transfer learning approaches. Across these implementations, the primary advantage consistently lies in improved data efficiency and faster early-stage discovery, an especially valuable feature in autonomous flow platforms where each experiment carries material, time, and energy costs.

However, transfer learning is not universally beneficial. Its success depends critically on the relatedness between source and target tasks, the quality of auxiliary datasets, and the ability of models to balance inherited bias with target-specific exploration. Negative transfer remains an under-recognized risk in chemical optimisation, particularly when reaction similarity is assumed rather than quantified. Unlike many benchmark problems in machine learning, chemical systems embed mechanistic complexity, sparse datasets, and heterogeneous variable types, making the principled definition of “task similarity” a central open challenge.

Future progress will likely depend on three converging developments. First, chemically meaningful representations, using informative descriptors, are needed to guide when and how knowledge should be transferred. Second, future approaches must combine models capable of learning from large and diverse reaction datasets with optimisation frameworks suitable for low-data experimental regimes.

Ultimately, transfer learning shifts the conceptual framework of reaction optimisation from isolated campaigns toward cumulative chemical learning. In this view, each optimisation is no longer an endpoint but a source of information that can inform future reactions, platforms, and scales. Realizing this vision will be key to transform self-driving laboratories from efficient search engines into genuinely knowledge-aware systems capable of navigating chemical space with increasing autonomy and insight.

## Author contributions

A. S. V., T.M., M.L. and F.-X. F. wrote the paper. F.-X. F. secured the funding.

## Conflicts of interest

There are no conflicts to declare.

## Data Availability

There are no data associated with this article.
